# Improved Toughness of PLA/PBAT/Modified Bamboo Powder Composites Through Interfacial Regulation

**DOI:** 10.3390/ma19050873

**Published:** 2026-02-26

**Authors:** Yonghuan Zhao, Yu Qi, Lei Song, Yuan Mei, Wenxiang Zhu, Yaofeng Zhu

**Affiliations:** 1 College of Materials and Science Engineering, Zhejiang Sci-Tech University, Hangzhou 310018, China; 2 Zhejiang Hailide New Materials Co., Ltd., Research Institute, Haining 312366, China

**Keywords:** bamboo–plastic composite, biodegradable, polydopamine, interfacial compatibilization, mechanical properties

## Abstract

The incorporation of abundant natural bamboo fiber (BF) into biodegradable polymers has emerged as a promising strategy to develop environmentally friendly materials. However, the poor interfacial compatibility between BF and biodegradable polymers has led to reduced performance, especially deteriorated toughness, and has limited the practical applications of bamboo–plastic composites. In this study, a compatible modifier, polydopamine (PDA), was employed to modify the surface of natural BF, and poly(lactic acid)/poly(butylene adipate-co-terephthalate) (PLA/PBAT) bamboo–plastic composites were fabricated via melt blending. And then, a commercial multifunctional compatibilizer (AX8900) was introduced to further enhance the interfacial compatibility and physical properties of the composite. After adding 20 wt% modified BF and 2 wt% compatibilizer, the composite exhibited a better notch impact strength (9.7 kJ/m^2^) than that filled with unmodified BF (3.2 kJ/m^2^), indicating a substantial enhancement. This work provides a novel approach to produce friendly biodegradable composites utilizing natural cellulose resources.

## 1. Introduction

As a new global pollutant, many studies have confirmed that microplastics are widely detected in oceans, soils, air, and in human bodies [1,2,3]. The degradation of conventional plastics, including polyolefins, is a protracted process that may take years, resulting in the sustained generation of microplastic contamination [4,5,6]. In response, the development of biodegradable materials has emerged as a critical strategy for addressing plastic pollution. Bio-based polymers, such as polylactic acid (PLA), polyhydroxyalkanoates (PHAs), and polybutylene succinate (PBS), offer a promising solution, as they can be fully mineralized by microbes into CO_2_ and water under specific conditions [7,8,9]. PLA is the most extensively employed biodegradable polymer in commercial applications, recognized for its excellent mechanical properties, biocompatibility, and safety profile [10]. However, it suffers from significantly low toughness, leading to failure in applications involving impact or bending [11,12]. A commonly employed strategy to improve the toughness of PLA entails blending it with another biodegradable polyester, PBAT, thereby forming a PLA/PBAT blend system [12,13]. For PLA/PBAT composites, fillers like calcium carbonate, talc, or starch are often incorporated into the resin system to improve the dimensional stability and reduce shrinkage.

The incorporation of mineral fillers such as calcium carbonate and talc can improve the shrinkage behavior, heat resistance, and cost-effectiveness of biodegradable polymers. However, these inorganic mineral fillers are non-biodegradable and require specific recycling protocols and end-of-life management, which contradicts the fundamental principle of biodegradable polymers. In contrast, BF is an abundant, renewable natural resource whose primary constituents, such as cellulose, are readily biodegradable under natural environmental conditions. The valorization of abundant, low-cost, and biodegradable natural fibers (e.g., bamboo, wood, straw) as fillers in polymers has provided a viable pathway to fabricate practical green composites [14,15]. The incorporation of bamboo flour (BF) has enabled the construction of a fully closed-loop biodegradable composite material system and significantly reduced the industrialization cost of degradable composites. However, natural plant fibers such as BF exhibit rough and irregular surface morphologies, and the industrially used BF typically has particle sizes over a range from 20 to 100 mesh, considerably larger than those of mineral fillers or micro/nano-scale fillers. Their irregular morphology and large particle size often lead to their poor dispersion and weak interfacial adhesion, which consequently deteriorates the mechanical performance of bamboo–plastic composites. In particular, the toughness and impact strength are substantially reduced, and are even below the intrinsic toughness of a neat polymer matrix. This phenomenon has been extensively documented in prior studies on PLA and various other bamboo–plastic polymer composites [16,17,18].

The attainment of superior dispersion and robust interfacial adhesion between natural fibers and a PLA/PBAT blend is crucial to prepare high-performance PLA/PBAT/BF composites [19,20]. Several surface modification techniques, including alkali treatment, acetylation, surface oxidation, and graft copolymerization, have been widely explored to enhance the dispersion of natural fibers in the polymer matrix [20,21,22]. A surface chemical modification effectively addresses the issue of weak interfacial bonding in wood–plastic composites and is regarded as a viable technical pathway. However, conventional chemical techniques are associated with significant drawbacks, notably the induction of surface damage and structural disruption to natural fibers, along with complex and labor-intensive procedures [22]. There is an urgent need to explore environmentally biological agents for fiber modification.

The use of dopamine hydrochloride (DA) as a bio-inspired surface modifier enables its self-polymerization into PDA at room temperature [23,24]. This results in a strongly adherent coating on various fillers, such as inorganic mineral powders, glass fibers and metal oxides, which significantly improves the dispersion and interfacial compatibility with a polymer matrix [25,26,27]. Much research has reported the successful fabrication of high-performance polymer composites using PDA-modified fillers. The PDA modification technique is a simple, green, and streamlined alternative to conventional chemical modification processing [28]. This approach has been applied to design biodegradable composites modified with natural fibers. Zhang et al. successfully functionalized bamboo powder pre-coated with PDA via grafting of a γ-glycidoxypropyltrimethoxysilane (KH560) silane coupling agent [29]. Notably, the resultant biodegradable polylactic acid (PLA)/modified bamboo powder composites exhibited markedly enhanced interfacial compatibility and superior physical properties. Haoran Zhai et al. incorporated BF surface modified with PDA/KH560 into PLA/PBAT blends [30]. The results demonstrated that the modified bamboo powder facilitated compatibility between the PLA and PBAT matrix phases, and simultaneously enhanced the interfacial compatibility between the bamboo fiber (BF) and the PLA/PBAT matrix through reactive compatibilization mechanisms. Consequently, the mechanical properties, thermal resistance, and hydrolysis resistance of the resulting composites were all significantly improved.

In the aforementioned study, although the modified natural fibers were shown to enhance the interfacial compatibility between PLA and PBAT, the intrinsic compatibility of the two polymers remained limited owing to the substantial disparities in their solubility parameters. Consequently, further improvement in the overall compatibility of the system is still attainable. By incorporating a compatibilizer bearing glycidyl methacrylate (GMA) or maleic anhydride grafts into PLA/PBAT-based wood–plastic or bamboo–plastic systems, the synergistic action of surface-modified natural fibers and supplementary compatibilizers can afford enhanced interfacial compatibility. Kehinde Olonisakin et al. first employed a steam treatment combined with myristic acid to modify the surface of bamboo powder, and subsequently introduced epoxy soybean oil as an interfacial compatibilizer into a PLA/PBAT/modified bamboo powder blend system [19]. Its characterization, based on interfacial modulus measurements, glass transition temperature analysis, and fracture surface morphology, revealed that the multi-level interfacial regulation mechanism arising from the concurrent application of surface modification and compatibilizer addition delivers superior interfacial compatibilization performance.

In this study, PDA-modified BF was first designed and subsequently incorporated into a PLA/PBAT blending. A commercially available compatibilizer, ethylene–methyl acrylate–glycidyl methacrylate copolymer (E-GMA, commercial name AX8900), was introduced to establish chemical bridging between the modified BF and the PLA/PBAT blending. Based on multiple interfacial regulation mechanisms, a high-toughness PLA/PBAT resin-based composite was successfully fabricated, resulting in a bamboo–plastic composite with simultaneously enhanced strength and toughness. This study provides a new way for the design and preparation of biodegradable wood–plastic composites.

## 2. Experiments and Characterization

### 2.1. Materials

PLA pellets (extrusion grade) were purchased from Hisun Biomaterials Co., Ltd. (Taizhou, China), with a density of 1.25 g/cm^3^, a melt index of 5~9 g/10 min tested at 190 °C/2.16 kg, and a melting temperature of 165 °C. PBAT pellets (extrusion grade) were supplied by Huafeng Co., Ltd. (Wenzhou, China), with a density of 1.20~1.25 g/cm^3^, a melt index of 2.5~4.5 g/10 min tested at 190 °C/2.16 kg, and a melting temperature of 120 °C. Natural BF (100 mesh) was obtained from Qingzhuyuan Co., Ltd. (Nanchang, China). Dopamine hydrochloride (analytical purity), Tris(hydroxymethyl)aminomethane (Tris, 99%), and deionized water were purchased from Macklin Biochemical Co., Ltd, Shanghai, China. Ethylene–methyl acrylate–glycidyl methacrylate terpolymer (E-GMA) (Commercial grade: AX8900) was supplied by SK Chemicals (Seongnam-si, Republic of Korea). It had a density of 0.94 g/cm^3^, a melt index of 4~8 g/10 min, and an EMA content of 8%.

### 2.2. Preparation of Polydopamine-Modified Bamboo Flour (MBF)

A total of 20 g of BF (100 mesh) was dried at 80 °C in a vacuum for 12 h, and then dispersed in 500 mL of deionized water. The mixture was stirred uniformly at 25 °C, and the pH of the system was adjusted to 8.5 using Tris buffer. After stirring for 30 min, 3 g dopamine hydrochloride was added, and the reaction was allowed to proceed for 24 h. The resulting product was repeatedly washed, filtered, and dried to obtain polydopamine-modified BF, denoted as MBF.

### 2.3. Preparation of PLA/PBAT Bamboo–Plastic Composites

**Figure 1:**
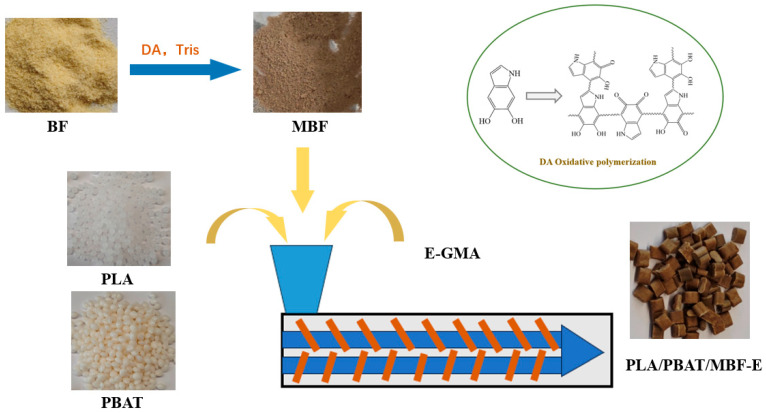
Schematic diagram of PLA/PBAT/MBF-E composite preparation process.

PLA/PBAT (weight ratio of 6:4) composite pellets were prepared via twin-screw extrusion. Based on a constant 20 wt% BF loading, composites filled with unmodified BF and MBF were prepared, designated as PLA/PBAT/BF and PLA/PBAT/MBF, respectively. Furthermore, the PLA/PBAT/MBF composite was melt-blended with the compatibilizer E-GMA (AX8900), resulting in a composite named PLA/PBAT/MBF-E. The experimental formulations of bamboo–plastic composite materials are shown in Table 1. The temperature of the twin-screw extruder (Nanjing Ruiya Extruder Co., Ltd., Nanjing, China, T35 model) was set at 140 °C, 170 °C, 175 °C, 175 °C, 175 °C, and 170 °C. The preparation process of the composite material is illustrated in Figure 1.

### 2.4. Characterization

The color changes in the BF were observed using an optical camera (D6, Nikon, Tokyo, Japan), and the microscopic morphological characteristics were investigated with a scanning electron microscope (SEM, JSM-5610LV, JEOL, Tokyo, Japan). Fourier transform infrared (FTIR) spectroscopy was employed to investigate the chemical structure of BF before and after modification over a range from 500 to 4000 cm^−1^. XRD was used to characterize the changes in the crystalline structure of BF. The diffraction patterns were recorded over a 2θ range of 5° to 50°.

The fracture surface morphologies of PLA/PBAT and various bamboo–plastic composites were examined using an SEM with an acceleration voltage of 10KV. All the specimens were gold-sputtered before analysis to ensure sufficient conductivity. FTIR spectroscopy (5700, Nicolet, Waltham, MA, USA) was employed to probe the structural changes, for which the samples were prepared as compressed pellets. The scanning range was 400~4000 cm^−1^. The mechanical properties of the PLA/PBAT bamboo–plastic composites were investigated with an Instron universal testing machine (5560, Instron, Norwood, MA, USA). The load cell had a capacity of 2000 N, and the testing standard followed GB/T 1040.1-2018. The tensile properties (tensile strength and elongation at break) were tested at a speed of 20 mm/min. Standard test specimens were prepared for testing by injection molding the composite pellets. The toughness of the materials was determined using an impact tester by measuring the notched Charpy impact strength. The tester model was WKT-XJ6001 and the hammer head mass was 5.5 J, operated according to the standard GB/T 9343-2008. Differential scanning calorimetry (DSC, 214, Netzsch, Bavaria, Germany) was employed to analyze the thermal behavior, and the samples were subjected to a thermal cycle between 30 °C and 200 °C at a scan rate of 10 °C/min. All the samples were rapidly heated to 200 °C and held for 2 min. The cooling curve was obtained by cooling down to 25 °C at a rate of 10 °C/min, and then the heating curve was obtained by heating up from 25 °C to 200 °C at the same temperature rate. The thermal stability of the PLA/PBAT bamboo–plastic composites was evaluated using a Pyris Diamond thermogravimetric analyzer (TGA) from PE Company in MA, USA. The measurements were conducted under a nitrogen atmosphere by heating the samples from 25 °C to 700 °C at a constant rate of 20 °C/min.

## 3. Results and Discussion

### 3.1. The Morphology of BF and MBF

**Figure 2:**
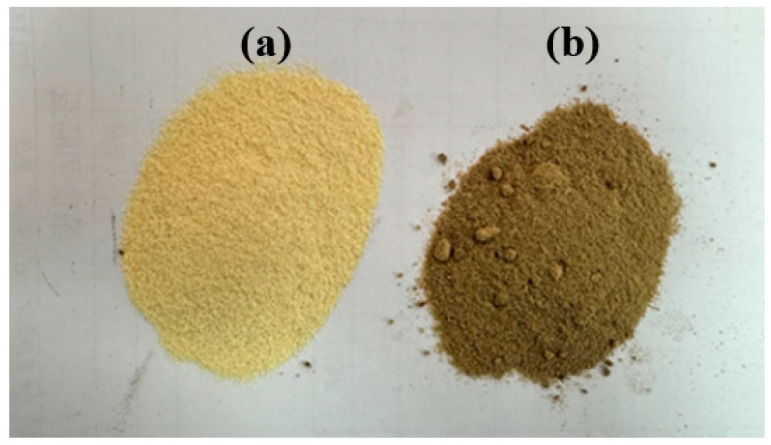
Optical photos of BF (**a**) and MBF (**b**).

**Figure 3:**
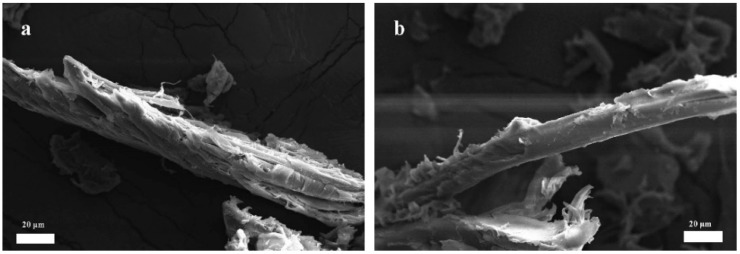
SEM images before and after BF modification: BF (**a**); MBF (**b**).

Figure 2 presents optical images of BF before and after modification. As can be observed from the figure, the natural BF showed a light yellow color, which transitioned to a grayish-brown after modification with PDA [31,32]. The oxidative self-polymerization of DA on the BF surface formed a PDA coating, as shown in Figure 3. The DA polymerization process occurred simultaneously on the outer surface and inner pore surface of BF particles. Therefore, it was possible to achieve sufficient encapsulation of complex morphologies. The PDA layer formed reduced the mutual embedding and adhesion caused by the rough surface of the natural BF. The sharp edges and grooves on the surface of BF were effectively encapsulated by the PDA coating, resulting in a significantly smoother and more rounded overall morphology. The successful deposition of PDA was evidenced by the distinct color change to grayish-brown [19,33].

### 3.2. Structural Analysis of BF and MBF

**Figure 4:**
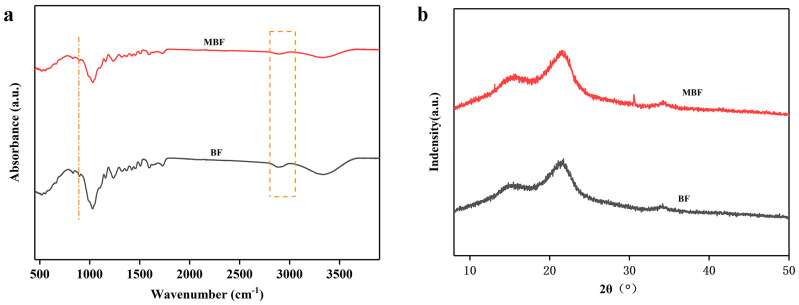
Structural characterization before and after BF modification: (**a**) FTIR; (**b**) XRD.

Figure 4a shows the FTIR spectra of BF and MBF. As presented in the figure, the peak patterns of BF and MBF were basically consistent. The prominent absorption peak at 1030 cm^−1^ was caused by the stretching vibration of C-O bonds in the aromatic esters (from cellulose, hemicellulose, and lignin) and alcohols [34,35]. The broad absorption peak near 3400 cm^−1^ corresponded to the stretching vibrations of hydroxyl (-OH) and amino (-NH) groups in the BF structure. The peaks in the region of approximately 1500–1600 cm^−1^ were attributed to the overlap of lignin from the BF and the aromatic rings of PDA. In the range of about 1040–1300 cm^−1^, a slight enhancement in the absorption bands was observed, which corresponded to the C-N and C-O stretching vibration absorptions of PDA. The formation of a PDA coating on the BF surface led to a reduction in the characteristic peak intensities of the MBF [36,37].

Figure 4b displays the XRD patterns of BF before and after modification. The diffraction peaks near 17.5°, 22°, and 36° corresponded to the (001), (002), and (040) crystal planes of natural cellulose [38,39]. There was no significant change in the position or shape of the diffraction peaks between the BF and MBF. Similarly, no obvious alterations were observed in the position or shape of the diffraction peaks in the XRD curves of BF and MBF. This indicates that PDA modification does not destroy the internal crystalline structure of natural cellulose. Compared with the common NaOH alkali treatment modification, the mild alkaline conditions at room temperature for DA self-polymerization were gentler [40]. This approach was advantageous for the subsequent applications, as it avoided etching or damaging the BF surface while simultaneously retaining the complete fiber structure, which was crucial for its role as a filler and reinforcement in resins.

### 3.3. FTIR of PLA/PBAT and Composites

**Figure 5:**
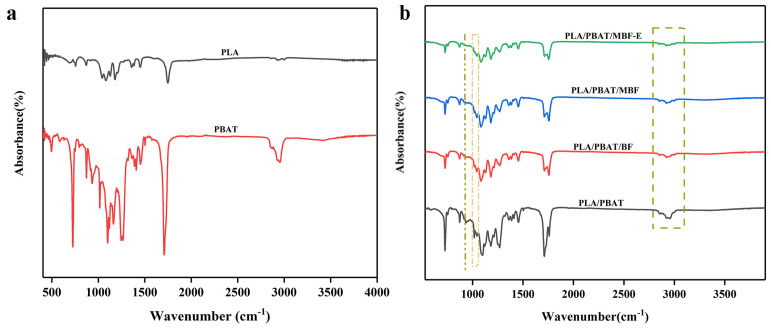
FTIR of PLA/PBAT (**a**) and various composites (**b**).

**Figure 6:**
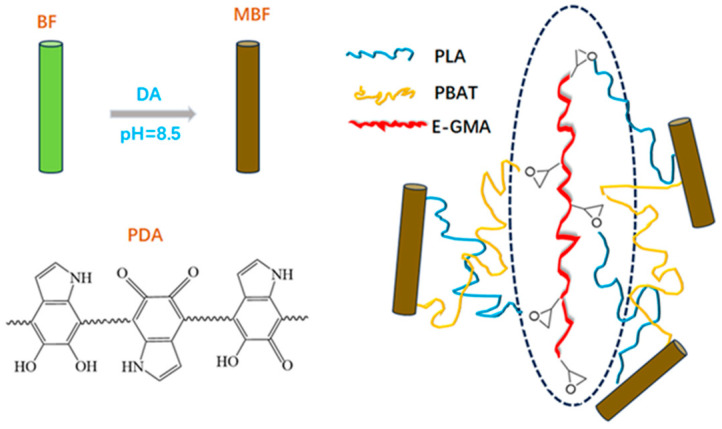
Schematic diagram of the reaction mechanism of the PLA/PBAT/MBF-E composite.

The infrared spectra of PLA/PBAT blends and bamboo–plastic composites are shown in Figure 5. As shown in Figure 5a, the characteristic peaks of PBAT included: a C=O stretching vibration peak at 1710~1715 cm^−1^; aromatic ring feature peaks at 720~730 cm^−1^; and asymmetric and symmetric stretching vibrations of methylene groups at 2950 cm^−1^ and 2860 cm^−1^, respectively [41]. The peak of PLA corresponded to the carbonyl stretching vibration observed at 1745–1750 cm^−1^, which appeared at a higher wavenumber compared to that of PBAT [42]. Additionally, a very strong and broad composite absorption band was observed in the range of 1080–1180 cm^−1^, representing another characteristic feature of polyesters. It can be observed from Figure 5b that the band primarily resulted from the combined asymmetric and symmetric stretching vibrations of the C–O–C groups in the ester bonds. After the addition of BF, the C–O stretching vibration peak at 1030 cm^−1^ become stronger and broader, indicating the substantial presence of cellulose [43]. In the PLA/PBAT/MBF composite, hydrogen bonding interactions occurred between the functional groups of PDA and the ester groups of the PLA/PBAT blend, resulting in a slight shift and broadening of the C=O absorption peak. Upon the introduction of E-GMA, the characteristic peak of the epoxy group at 910 cm^−1^ became weaker. The multi-epoxy-functional E-GMA, as a molecular bridge, engaged in ring-opening reactions with the terminal groups of both the PDA layer and the PLA/PBAT to form covalent bonds, and the schematic diagram of the reaction mechanism of the PLA/PBAT/MBF-E composite is shown in Figure 6. It established a robust “polymer–compatibilizer–coupling agent–filler” interfacial bridge, which significantly enhanced the cohesion among the various components of the bamboo–plastic composite.

### 3.4. SEM of PLA/PBAT and Composites

**Figure 7:**
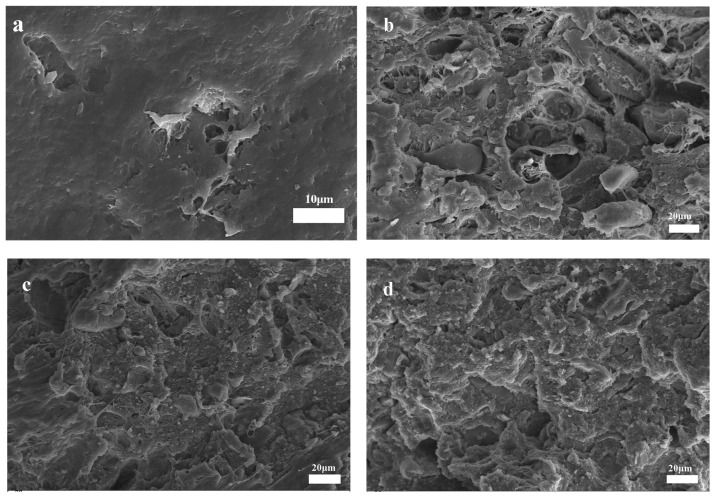
SEM: (**a**) PLA/PBAT; (**b**) PLA/PBAT/BF; (**c**) PLA/PBAT/MBF; (**d**) PLA/PBAT/MBF-E.

Figure 7 exhibits the SEM micrographs illustrating the fracture surface morphologies of PLA/PBAT and various bamboo–plastic composites. As observed in Figure 7a, obvious interface holes were evident on the fracture surface of the neat PLA/PBAT blend. This was attributed to the relatively large difference in the solubility parameters between the PLA and PBAT. Figure 7b reveals the presence of BF agglomerates on the fracture surface of the PLA/PBAT/BF composite. The unmodified BF was prone to agglomeration, leading to a significant number of voids and defects at the interface with the PLA/PBAT blend matrix. As shown in Figure 7c, an obvious improvement in the dispersion of MBF within the matrix and its interfacial adhesion can be observed, where the bamboo fibers are tightly encapsulated by the PLA/PBAT resin. This indicates that the PDA coating effectively promoted interfacial compatibility between the MBF and polymer blends. Meanwhile, the high surface activity of PDA played a role in interface coupling and mechanical anchoring, significantly improving the interface compatibility between the PLA and PBAT. Following the incorporation of the multi-epoxy-functionalized reactive compatibilizer E-GMA, the interfacial adhesion of the PLA/MBF/E-GMA composite was substantially improved, as presented in Figure 7d. The E-GMA acted as a chemical bridge between the MBF and the PLA/PBAT matrix. The strong covalent molecular bridging facilitated interfacial compatibilization, thereby establishing a robust interfacial bonding effect [44,45].

### 3.5. Mechanical Properties of PLA/PBAT and Composites

**Figure 8:**
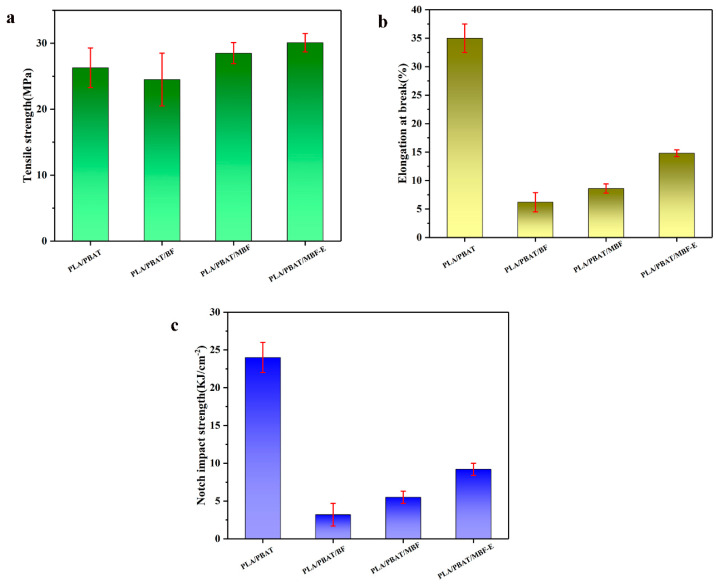
Mechanical properties: (**a**) tensile strength; (**b**) elongation at break; (**c**) notch impact strength.

The mechanical properties of various composite materials are illustrated in Figure 8. It was found that the bamboo–plastic composites exhibited improved tensile strength compared to the neat PLA/PBAT. This phenomenon can be attributed to the fibrous BF serving as a reinforcing phase, which transferred the load stresses during tensile and bending deformations [46,47]. The tensile strength of PLA/PBAT/MBF was 29.4 MPa, which was higher than that of PLA/PBAT/BF.

Conversely, the bamboo–plastic composites exhibited a negative effect on the notched impact strength, which is same as the adverse contribution commonly associated with other natural fiber-filled polymers [48,49,50]. Despite its inherent one-dimensional fibrous structure, natural BF has a low aspect ratio and a rough, irregular surface, which typically compromise the impact strength upon incorporation, as demonstrated in prior studies [51,52,53]. The impact strength of bamboo–plastic composites was enhanced after the modification of BF. Compared to the PLA/PBAT/BF composite, the PLA/PBAT/MBF-E composite displayed enhanced elongation at break and impact strength, reaching 13.5% and 9.7 kJ/m^2^, respectively. This may primarily be attributed to two key factors: (1) the well-dispersed PDA-modified MBF effectively reduced the stress concentration within the matrix; and (2) the strong interfacial adhesion significantly increased the energy required for crack propagation.

### 3.6. Thermal Properties of PLA/PBAT and Composites

**Figure 9:**
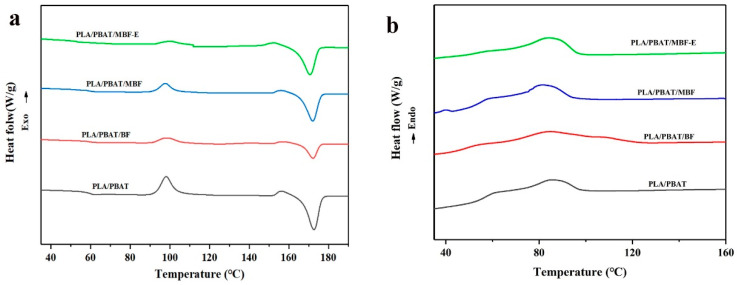
DSC of various composites: (**a**) heating curve; (**b**) cooling curve.

The DSC results for the PLA/PBAT and bamboo–plastic composites are as shown in Figure 8. As illustrated in Figure 9a, a cold crystallization peak and a melting peak were observed in the PLA/PBAT blends, which could be due to the promoted heterogeneous nucleation and the altered chain segment mobility of PLA upon the incorporation of PBAT [54]. In Figure 9b, it can be seen that the incorporation of the unmodified BF inhibited the crystallization of the PLA/PBAT bamboo–plastic composites. Owing to the presence of numerous hydrogen bonds in unmodified BF, the addition of 20% BF induced agglomeration in the polymer matrix, which restricted the relaxation of PLA molecular chains [55,56]. This disruption hindered the rearrangement and nucleation ability of the molecular chains, which impaired the crystallization capacity. Moreover, the improved dispersion of MBF enhanced the nucleation efficiency of the composite. Both the cold crystallization peak in Figure 9a and the crystallization peak observed on the cooling curve in Figure 9b exhibit a concurrent recovery and enhancement.

After addition of the compatibilizer E-GMA, the intensified interfacial reactions among the MBF, PLA, and PBAT reduced the structural defects of the composite. There also existed a competitive mechanism between the interfacial compatibility and chain branching that influenced the crystallization behavior. In Figure 9a, the cold crystallization temperature increases, whereas the peak intensity decreases significantly. In Figure 9b, the crystallization temperature shows only a slight elevation. The high content of EMA promoted interfacial compatibility among the PLA, PBAT, and MBF. However, the simultaneously high reactivity also facilitated the chain extension and branching of PLA and PBAT to some extent, as well as possible reactions at the chain ends [56,57]. The molecular architecture contributed to enhanced toughness of the resulting composites, a feature of considerable significance in bamboo–plastic composites.

**Figure 10:**
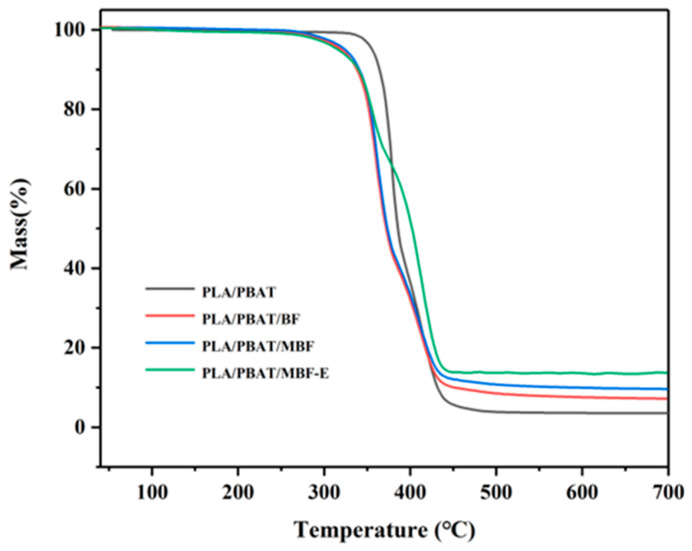
TGA curves of PLA/PBAT and composites.

The thermal degradation properties of the composites were evaluated by thermogravimetric analysis (TGA). The TGA curves are presented in Figure 10. The PLA primarily underwent degradation between 300 and 400 °C, and the PBAT showed a main weight loss in the range of 350 to 400 °C [58,59]. For the bamboo–plastic composites, the hemicellulose component within the BF began to decompose at 200~260 °C [60], which was the thermally weak component. Upon the addition of the compatibilizer E-GMA, the intensified interfacial reactions among the MBF, PLA, and PBAT reduced the structural defects of the composite, resulting in the initial degradation temperature of the entire composite. Moreover, the poor compatibility of the PLA/PBAT/BF composite caused voids and defects at the interface, which served as initiation sites for thermal degradation. As a result, the PLA/PBAT/BF composite exhibited compromised thermal stability. The higher initial degradation temperature observed for the PLA/PBAT/MBF was attributed to the improved interfacial adhesion. Compared to the PLA/PBAT/BF, the PLA/PBAT/MBF composite displayed more excellent thermal stability when the temperature was elevated beyond 350 °C, as shown in Table 2. The thermal stability of the PLA/PBAT/MBF-E was dramatically improved because of the improved interface structure. Meanwhile, the enhanced carbonization behavior of the composite materials led to a markedly greater residual carbon yield of about 14.5% relative to the previously described specimens. This can be explained by the fact that strong interfacial adhesion facilitates the formation of a continuous and dense carbon layer, which reduces the transfer of heat and oxygen and suppresses the thermal decomposition of a system.

## 4. Conclusions

In summary, PLA/PBAT/MBF bamboo–plastic composites were successfully prepared through a simple and feasible process of melt blending and extrusion. The surface modification of BF using PDA effectively enhanced the interfacial adhesion within the composite, while the introduction of the E-GMA compatibilizer further improved the interfacial bonding among the modified bamboo flour (MBF), PLA, and PBAT, resulting in a bamboo–plastic composite with well-balanced comprehensive properties. Compared with the PLA/PBAT/BF composite, the notch impact strength of the PLA/PBAT/MBF-E composite significantly increased from 3.2 KJ/m^2^ to 9.7 KJ/m^2^. This study opens up a new route for the utilization of abundantly available natural bamboo fibers in the development of composite materials. Subject to the prohibitive costs of dopaminergic precursors and the extended reaction time, the technical viability of this synthetic modification strategy remains insufficiently mature in industrialized production. However, the resulting biodegradable bamboo–plastic composites are environmentally friendly, and merit further exploration of their industrial applications in the future.

## Figures and Tables

**Table 1:** The experimental formulation of bamboo–plastic composite materials.

	PLA/PBAT	PLA/PBAT/BF	PLA/PBAT/MBF	PLA/PBAT/MBF-E
PLA	60	60	60	60
PBAT	40	40	40	40
BF	/	20	/	/
MBF	/	/	20	20
E-GMA	/	/	/	2

**Table 2:** TGA data for PLA/PBAT and composites.

Sample	PLA/PBAT	PLA/PBAT/BF	PLA/PBAT/MBF	PLA/PBAT/MBF-E
T_5_ (°C)	354.2	322.8	323.2	325.4
T_50_ (°C)	385.5	372.1	373.6	402.6
Residual carbon (%)	0.8	6.7	9.4	14.5

## Data Availability

The original contributions presented in this study are included in the article. Further inquiries can be directed to the corresponding author.
